# Interactive Effects of Elevated [CO_2_] and Drought on the Maize Phytochemical Defense Response against Mycotoxigenic *Fusarium verticillioides*

**DOI:** 10.1371/journal.pone.0159270

**Published:** 2016-07-13

**Authors:** Martha M. Vaughan, Alisa Huffaker, Eric A. Schmelz, Nicole J. Dafoe, Shawn A. Christensen, Heather J. McAuslane, Hans T. Alborn, Leon Hartwell Allen, Peter E. A. Teal

**Affiliations:** 1 Mycotoxin Prevention and Applied Microbiology Research Unit, National Center for Agricultural Utilization Research, United States Department of Agriculture, Agricultural Research Service, 1815 N University St, Peoria, Illinois, 61604, United States of America; 2 Chemistry Research Unit, Center of Medical, Agricultural, and Veterinary Entomology, United States Department of Agriculture, Agricultural Research Service, 1600 SW 23^rd^ Drive, Gainesville, Florida, 32608, United States of America; 3 Department of Nematology and Entomology, University of Florida, Gainesville, Florida, 32610, United States of America; The University of Wisconsin - Madison, UNITED STATES

## Abstract

Changes in climate due to rising atmospheric carbon dioxide concentration ([CO_2_]) are predicted to intensify episodes of drought, but our understanding of how these combined conditions will influence crop-pathogen interactions is limited. We recently demonstrated that elevated [CO_2_] alone enhances maize susceptibility to the mycotoxigenic pathogen, *Fusarium verticillioides* (*Fv*) but fumonisin levels remain unaffected. In this study we show that maize simultaneously exposed to elevated [CO_2_] and drought are even more susceptible to *Fv* proliferation and also prone to higher levels of fumonisin contamination. Despite the increase in fumonisin levels, the amount of fumonisin produced in relation to pathogen biomass remained lower than corresponding plants grown at ambient [CO_2_]. Therefore, the increase in fumonisin contamination was likely due to even greater pathogen biomass rather than an increase in host-derived stimulants. Drought did not negate the compromising effects of elevated [CO_2_] on the accumulation of maize phytohormones and metabolites. However, since elevated [CO_2_] does not influence the drought-induced accumulation of abscisic acid (ABA) or root terpenoid phytoalexins, the effects elevated [CO_2_] are negated belowground, but the stifled defense response aboveground may be a consequence of resource redirection to the roots.

## Introduction

In 2015 the global average concentration of carbon dioxide ([CO_2_]) in the atmosphere reached a record high of 400 μmol CO_2_ mol^-1^ air and if current trends continue by the end of this century it could even surpass 800 μmol CO_2_ mol^-1^ air [1]. Rising [CO_2_] is largely responsible for changes in our climate including increased temperatures and altered precipitation patterns. These changes in weather patterns will ultimately influence crop productivity and are predicted to be particularly detrimental to summer crops, such as maize, which will likely experience severe episodes of drought. Maize (*Zea mays*) represents an essential part of the world’s grain food and feed supply, and the majority of the maize cropping systems depends on natural precipitation [2]. Maize uses the C_4_ photosynthetic mechanism which is not limited by [CO_2_], and therefore, yields will only benefit from rising [CO_2_] under conditions of drought when the indirect effect of reduced stomatal conductance enhances the plants water-use efficiency allowing photosynthesis to continue despite limited water conditions [3–5]. Nevertheless, in addition to abiotic stress, plant diseases and insect pests are also major limiting factors of maize productivity, yield, and quality; however, our understanding of how the combination of both elevated [CO_2_] and drought will affect maize susceptibility to biotic stressors is limited.

The mycotoxigenic fungal pathogen, *Fusarium verticillioides* (*Fv*) not only reduces the maize yield by causing rot in all parts of the plant [6,7] but also produces carcinogenic polyketide-derived mycotoxins termed fumonisins which render harvested grain unsafe for human or animal consumption. Mycotoxins, such as fumonisins, are among the top food safety concerns with regard to climate change [8] because environmental conditions predicted for the future are important factors that contribute to fumonisin contamination. Warmer temperatures increase evapotranspiration further intensifying drought which has been shown to correlate with *Fv* disease development and enhance fumonisin accumulation in grain [7,9,10].

Recently, we demonstrated that elevated [CO_2_] (800 μmol CO_2_ mol^-1^ air) also enhances maize susceptibility to *Fv* infection, but the increase in fungal biomass did not correspond with greater fumonisin levels resulting in an overall reduction in fumonisin per unit fungal biomass [11]. Following *Fv* inoculation, the accumulation of maize soluble sugars, free fatty acids, lipoxygenase (LOX) transcripts, jasmonic acid (JA) and salicylic acid (SA) phytohormones, and terpenoid phytoalexins was dampened at elevated [CO_2_] [11]. An influx of fatty acid substrate is essential for the burst of JA that initiates the defense signaling process [12]. JA and other oxylipins are synthesized from free fatty acids through the LOX pathway [13–16]. The fatty acids are oxidized by LOX enzymes at either the 9 or 13 carbon position to produce 9-LOX or 13-LOX oxylipins, respectively. The phytohormone JA is a 13-LOX oxylipin derived from linolenic acid. The defense related functions of 9-LOX metabolites are not well characterized, but they have been implicated in the stimulation of mycotoxin production [14,17,18]. Elevated [CO_2_] appears to effect both 9- and 13-LOX oxylipin biosynthesis at the level of fatty acid substrate supply and LOX-gene transcription [11]. Lower concentrations of defensive phytochemicals, such as the zealexin and kauralexin terpenoid phytoalexins, due to compromised JA biosynthesis and signaling is consistent with increased *Fv* proliferation. Additionally, a dampened response of 9-LOX metabolites could reduce the ratio of fumonisin per *Fv* biomass [11].

Elevated [CO_2_] similarly enhances C_3_ crop (i.e. soybean, tomato) susceptibility to herbivory by compromising LOX-gene transcription, JA biosynthesis and JA-dependent antiherbivore defenses. However, in C_3_ crops JA regulated defenses appeared to be compromised by an antagonistic boost in SA production, which does not occur in maize [11,19–21]. Furthermore, the effects of elevated [CO_2_] were negated when soybean plants were simultaneously exposed to drought stress.

Whether drought will negate the effects of elevated [CO_2_] on maize susceptibility to *Fv* is unknown, and it is unclear what the interactive effects of elevated [CO_2_] and drought will do to fumonisin levels. Although individual stress responses display measurable specificity, plants are frequently simultaneously challenged by several stress factors resulting in the activation of multiple signals that engage in cross-talk and alter individual responses. For example, abscisic acid (ABA), which typically functions in mediating responses against abiotic stress such as drought [22], can have a synergistic effect on JA. Furthermore, the induction of ABA with drought has been shown to promote resistance against some fungal pathogens [23–27]. However, if drought does not negate the effects of elevated [CO_2_] and fumonisin production is stimulated, future food safety issues could become more severe.

In this study we investigated the combined impact of elevated [CO_2_] and drought on maize susceptibility to *Fv* infection and fumonisin contamination using the maize stalk infection assay which we previously demonstrated to be representative of ear infection [11]. To evaluate the interactive effects of elevated [CO_2_] and drought on the maize defense response against *Fv*, we compared the concentration of carbohydrates, starch, proteins, free fatty acids, phytohormones, benzoxazinoids and terpenoid phytoalexins in both infected and control stalk tissues under individual and simultaneous abiotic stress treatments (elevated [CO_2_] and drought). Furthermore, since terpenoid phytoalexins were recently shown to accumulate in maize roots [28], we evaluated the effects of [CO_2_] on the accumulation of root terpenoid phytoalexins in response to drought. However, since it was initially unclear if the effects of elevated [CO_2_] on JA signaling persisted in the belowground tissues, the effects of elevated [CO_2_] and drought on the maize root defense response to *Diabrotica balteata* (rootworm) larval feeding was also evaluated. Finally, we discuss the potential implications of our findings on the future of maize grain security and safety under expected climate change conditions.

## Materials and Methods

### Experimental Design of *F*. *verticillioides* maize stalk inoculations

A total of 32 pots containing four *Zea mays* (maize) plants were grown in each of two environmental Conviron E15 (Pembina, ND, USA) growth chambers controlled at two different [CO_2_]: 400 μmol CO_2_ mol^-1^ air (1x[CO_2_]) and the other at 800 μmol CO_2_ mol^-1^ air (2x[CO_2_]). Using a complete randomized design in each chamber, a drought (-H_2_O) treatment was imposed on a subset of 16 pots at 25 d post by withholding water for 5 d. At 30 d post sowing, the +H_2_O and -H_2_O maize were inoculated by slitting the stem and injecting 100 μL of either a 1x10^6^ *F*. *verticillioides* (*Fv*) (Northern Regional Research Laboratory [NRRL] stock no. 7415) spores mL^-1^ (+*Fv*) or a control 0.1% Tween 20 solution (-*Fv*). Methods regarding preparation of fungal inoculum and stalk inoculation have previously been described [29]. Four biological replicates were designated and inoculated per treatment. Each biological replicate was composed of eight plants from two independent pots. Unless otherwise stated, the treated stem tissue was collected 2 d post inoculation by removing the area around the inoculation site. The experiment was repeated four times and the chambers were switched between repeated experiments. Comparisons between plant defense responses at 1x[CO_2_] and 2x[CO_2_] with irrigated (+H_2_O) have been published separately [11]. This manuscript focuses on the combined effects of elevated [CO_2_] and drought on maize susceptibility to *Fv* infection and fumonisin contamination in maize.

### Plant material and growth conditions

*Zea mays* var. *Golden Queen*, a sweet corn commonly grown for fresh market throughout Florida, (Southern States Cooperative, Inc., Richmond, VA, USA) was used for the reported experiments. Four plants were grown per pot (10.5 cm x 10.5 cm x 12 cm high), filled with MetroMix 200, (Sun Gro Horticulture Distribution, Inc, Bellevue, WA, USA) supplemented with 14-14-14 Osmocote (Scotts Miracle-Gro, Marysville, OH, USA). Other than the difference in [CO_2_], all other conditions of the growth chambers were identically controlled at 28°C day/25°C night, 500 μmol m^−2^ s^−1^ photosynthetic photo flux density 12 h photoperiod and between 50 and 60% relative humidity. Environmental conditions were monitored and controlled as previously described [29]. The plants were watered daily and received bi-weekly nutrient supplement with soluble Peters 20-20-20 (The Scotts Company, Marysville, OH, USA). During the drought treatment, the irrigated (+H_2_O) plants received only water, no nutrient supplement.

### Drought Treatment

A 5 d treatment of -H_2_O was chosen because it was the point at which maize plants at 1x[CO_2_] started to exhibit visually detectible drought stressed phenotypes such as leaf blade curling. Plants at 2x[CO_2_] still appeared relatively non-stressed. However, to verify that this was the point at which maize was benefitting from elevated [CO_2_] by increased water-use efficiency, the stomatal conductance for water vapor (g_s_), soil water content and photosynthetic CO_2_ assimilation rate (Pn) were measured. Stomatal conductance for CO_2_, g_sc_ is g_sc_ = g_s_/1.6. Only g_s_ will be reported herein.

Gas exchange measurements for obtaining Pn and g_s_ were taken with an open-flow portable leaf photosynthesis system (Li-Cor 6400XT, Li-Cor Inc., Lincoln, NE, USA) equipped with the standard leaf chamber (6 cm^2^ of leaf area) and CO_2_ injection system (model 6400–01, Li-Cor Inc., Lincoln, NE, USA) adjusted to a constant [CO_2_] of 400 μmol CO_2_ mol air^–1^ (1x[CO_2_]) or 800 μmol CO_2_ mol air^–1^(2x[CO_2_]). The environmental conditions in the Li-Cor 6400 chamber were adjusted to match those of the growth chamber. Measurements were performed between 11:00 AM–1:00 PM on the fifth leaf from the bottom for four individual plants under each environmental condition.

Percentage of soil water content was measured using an EC-5 soil moisture sensor attached to the ProCheck sensor read-out system (Decagon Devices, Pullman, WA, USA) and verified using a gravimetric method [30]. Fresh weight soil samples were collected from each pot with a 3 cm diameter cork borer. Four biological replicates consisting of a pool of soil samples from two pots were collected for each of the conditions. The samples were dried in an oven for 4 d at 60°C, the dry weight was recorded and the percent water content was calculated. The difference in water availability was not adjusted for equivalence because it was due to a natural physiological response to the climatic treatment.

### Quantification of *Fusarium verticillioide*s (*Fv*) DNA and fumonisin in plant tissue

The amount of pathogen DNA relative to plant DNA was estimated at 2 d and 7 d post inoculation using quantitative real-time PCR (qRT-PCR) following methods previously described [29]. In summary, a five-fold dilution series of pure *Fv* DNA and pure maize DNA was used to generate standard curves by plotting the Cq values obtained by RT-PCR against the log([DNA]) [31]. These curves were then used to estimate quantities of each specific species DNA in the infected plant tissue. The amount of *Fv* DNA was determined using beta-tubulin (KC964147) specific primers: Fv_*TUB2*_F (5’-TGCTCATTTCCAAGATCCGCG-3’) and Fv_*TUB2*_R *(**5’-*gtagttgaggtcaccgtaggagg-3’). Plant DNA quantification was performed using elongation factor 1α (*Ef1α*) gene (NM_001112117) specific primers: Zm_*Ef1α*_F (5’-gcttcacgtcccaggtc-3’) and Zm_*Ef1α*_R (5’-ataggcttggtgggtatca-3’).

The Veratox fumonisin kit (Neogen, Lansing, MI, USA) and Veratox 3.0 for windows software was used to quantify the amount of fumonisin produced by *Fv* in maize stem tissue experiencing the abiotic stress treatments. The kit uses a direct enzyme-linked immunosorbent assay to determine the total fumonisins (B1, B2, B3) between the quantization rage of 1 and 6 μg g^−1^ and has been verified various grains including corn [32]. However, an additional validation was performed for maize stalk tissue by artificially contaminating samples with known quantities of fumonisins B1. The known quantity of fumonisin in a sample was plotted against the estimated quantity determined from using the kit. The r^2^ value obtained from the standards provided by the kit was 0.997, and the r^2^ value obtained from standards made in our lab mixed with maize stalk tissue was 0.956. Since the fumonisin levels were below detectable levels at 2 d post inoculation time point, the pathogen was allowed to establish for 7 d prior to analysis. For this longer treatment, drought was similarly imposed by withholding water for 5 d prior to application of pathogen, but 2 d after *Fv* inoculation (at which point the shorter 2 d experiment was terminated), the -H_2_O stressed plants were watered but then again water was withheld for the remainder of the experiment. As with the shorter experiment, four biological replicates consisting of a pool of eight stems were collected for analysis. Fumonisin was extracted from 0.25 g of ground tissue with 1 mL 70% methanol, diluted 5-fold and quantified following the kit manufacturers protocol.

### Phytochemical analyses of abiotic and biotic stressed maize tissues

To be able estimate the dry weights of tissue samples for phytochemical quantification purposes, a portion of the stem tissue just below the inoculated (+*Fv*) or control (-*Fv*) area was collected at the end of the 2 d pathogen infection experiment and the fresh and dry weight were used to estimate the average percentage of water within the stem tissues from the different treatments. Using this average percentage of water content, the dry weight could be estimated from the fresh weight of each sample so that the concentration of metabolites could be calculated on a dry weight basis. Thus, the differences in water content would not influence metabolite concentration comparisons.

The concentration of phytohormones (JA, SA, and ABA), primary metabolites and secondary metabolites was determined to evaluate the combined effects of 2x[CO_2_] and–H_2_O on the maize phytochemical response to +*Fv*. The quantification of soluble carbohydrates, starch, proteins, free fatty acids, JA, SA, MBOA, kauralexins, and zealexins in maize tissues was performed as previously described by Vaughan *et al*., (2014). Benzoxazinoid hydroxamic acids (DIMBOA-Glc and HDMBOA-Glc) were analyzed by HPLC as previously described [33,34]. Briefly, metabolites were extracted from lyophilized tissue in 98:2 methanol: acetic acid containing 50 μg mL^-1^ 2-benzoxazolinone which was used as an internal standard for quantification.

### Initial burst of phytohormones

To determine the interactive effects of 2x[CO_2_] and–H_2_O maize on phytohormone signaling, the initial burst of JA and SA was tracked by collecting tissue samples throughout a time course following +*Fv*. As above, maize stems were slit and inoculated with 100 μL of 1x10^6^ spores mL^-1^ *Fv*. Tissue samples were collected immediately after inoculation (time 0 min) and at 15 min, 30 min, 60 min. Four biological replicates each derived from two individual pooled plants were collected frozen in liquid N_2_ and stored at -80°C. Phytohormone extraction and quantification was then performed as previously described [29,35].

### Evaluation of maize root defense response

To evaluate the induction of root phytoalexins in response to drought, maize grown in potting mix was subjected to seven days of consecutive drought throughout which a subset of biological replicates were sacrificed for phytohormone analyses. As described above, drought treatment was imposed by withholding water. Each day at noon throughout the time course the soil volumetric water content (VWC) was measured using an EC-5 soil moisture sensor attached to the ProCheck sensor read-out system (Decagon Devices, Pullman, WA, USA), and the root mass of five biological replicates was collected. Each biological replicate represents the root mass from two plants grown in the same pot.

The generalist root herbivore of maize, *Diabrotica balteata* LeConte was used to assess differences in the root defense response to biotic stress under the variable abiotic stress conditions imposed. The same experimental design described above for stalk inoculation was used for the larval feeding experiments; however, only a single maize seedlings was grown per pot filled with Seramis clay granules which provided convenient access to the root tissue without causing additional damage to remove the root from the growth medium [28,36]. Drought treatment was similarly imposed by withholding water for 5 days (approximately 25% VWC) prior to the introduction of the biotic stress. Larvae were reared according the methods of [28,37]. Briefly, beetle eggs oviposited into containers with moistened cheesecloth and sterilized in sodium hypochlorite solution were placed on sprouted corn seeds and allowed to hatch. Ten 2^nd^ and 3^rd^ instar larvae were released into the clay substrate of V2 maize seedlings. The larvae were allowed to feed for 2 d prior to root recovery. Four biological replicates were collected per treatment. Each biological replicate consisted on the entire root mass from a single independent plant. Samples were frozen in liquid nitrogen, pulverized and analyzed as described above.

### Statistical analysis

Since there are multiple explanatory factors within these experiments, a 2x2 (1x[CO_2_]/2x[CO_2_] x +H_2_O/-H_2_O; 1x[CO_2_]/2x[CO_2_] x -*Fv* /+*Fv*) or 2x2x2 (1x[CO_2_]/2x[CO_2_] x +H_2_O/-H_2_O x -*Fv* /+*Fv*) full factorial analysis of variance (ANOVA) was performed to determine which factors contributed to differences. Significant factors and interactions have been listed at the top right corner of figures which contain multiple comparisons. If there was no interaction between the potential contributing factors, a pair wise student’s t-test or ANOVA followed by Tukey- Kramer honestly significant difference (HSD) test was performed on mean of the main effects to determine which were significantly different. However, if there was a significant interaction between all the factors, a Tukey- Kramer honestly significant difference (HSD) test was performed to determine differences between interaction means. Because the main contributing factors were different depending on the variable being analyzed, statistical differences were not always indicated in the figure above each mean bar unless there was a significant interaction between all the factors and the ANOVA comparisons were made between interaction means. Most of the figures display only comparisons between 1x[CO_2_] and 2x[CO_2_] metabolite concentrations under conditions of -H_2_O. Comparisons between metabolite concentrations of +H_2_O plants at 1x[CO_2_] and 2x[CO_2_] have previously been published [11]. However, statistical analyses were performed to compare -H_2_O data reported here with the previously published data [11].

For the time course experiments, differences between variable concentrations in plants at 1x[CO_2_] in comparison to 2x[CO_2_] were determined independently for each individual time point using a Student’s *t*-test. Separate analyses were used to compare variable means of +H_2_O and–H_2_O stressed plants at 1x[CO_2_] or 2x[CO_2_].

## Results

### Drought treatment is representative of conditions in which maize plants benefit from elevated [CO_2_] induced water conservation

**Fig 1:**
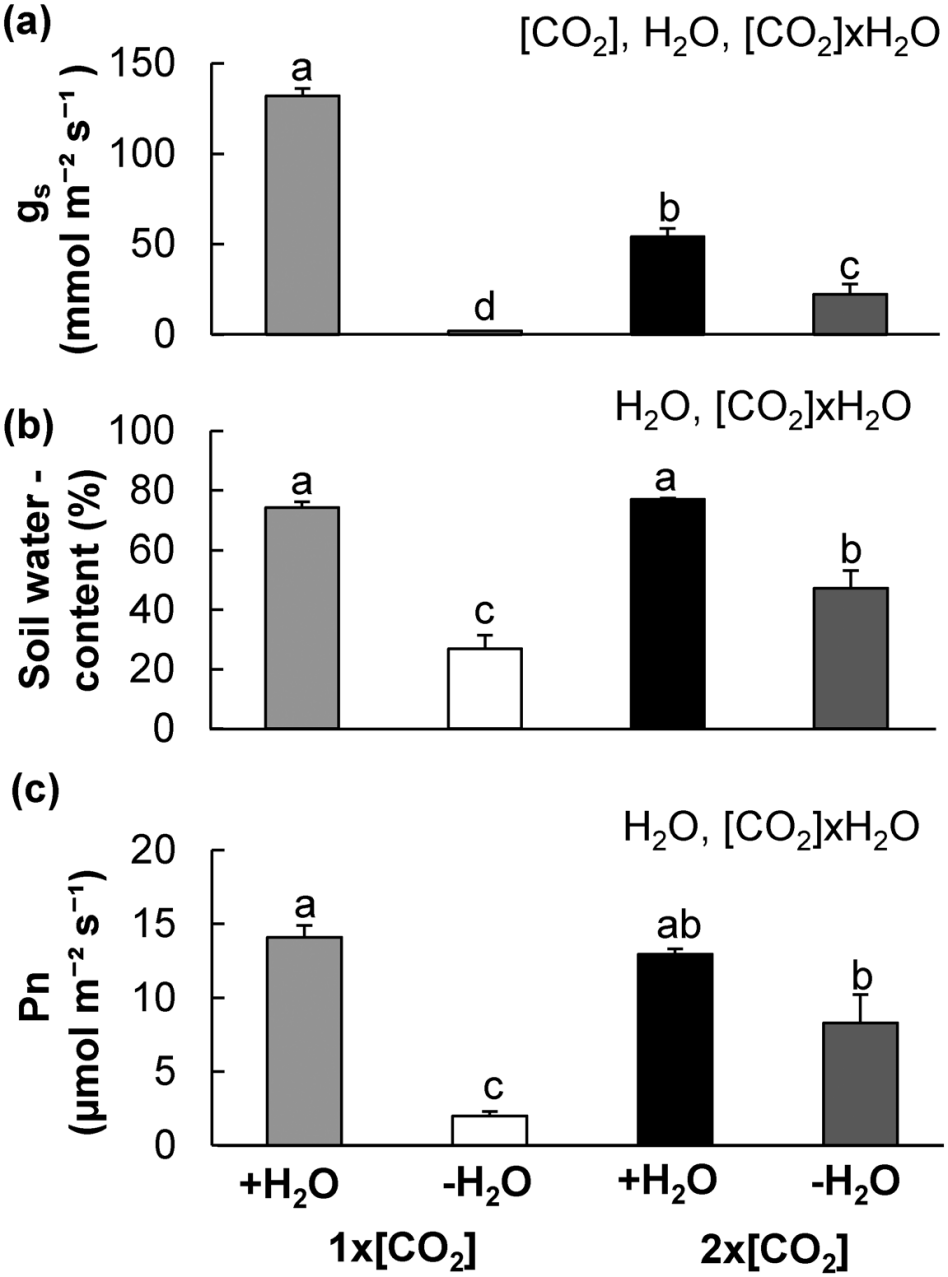
Drought treatment is representative of conditions in which maize plants benefit from elevated [CO_2_] induced water conservation. Drought treatment was imposed on one month old maize by withholding water for five consecutive days. Gas exchange measurements were then taken from the fifth leaf of 1x[CO_2_] (400 μmol CO_2_ mol^-1^ air) or 2x[CO_2_] (800 μmol CO_2_ mol^-1^ air) grown maize. The average (a) stomatal conductance (g_s_), (b) percent soil water content and (c) photosynthetic rate (Pn) was estimated for each set of plants with irrigation (+H_2_O) or drought (-H_2_O) treatment. The statistically significant main effects of differences determined by a 2x2 ([CO_2_]xH_2_O) analysis of variance (ANOVA) are indicated in the top right hand corner of each graph. Since the interaction between the contributing factors was significant, a Tukey- Kramer honestly significant difference (HSD) test was performed to determine differences between means. Letters above standard error of mean (SEM) bars indicate significant differences (n = 4, *P*<0.01).

Elevated [CO_2_] reduces g_s_ which can ameliorate drought stress by conserving water and enabling photosynthesis to continue for longer during episodes of drought [4,38,39]. To first confirm that the drought treatment imposed stress on both sets of plants but also exemplified the beneficial interactions between elevated [CO_2_] and drought, the g_s_, soil water content, and Pn were measured. There was a significant interaction between [CO_2_] and watering regime (H_2_O) for all three variables examined (Fig 1). The g_s_ was approximately 60% lower in leaves of plants at 2x[CO_2_] in comparison to 1x[CO_2_]+H_2_O. Treatment of -H_2_O also reduced g_s_ in plants at both [CO_2_], but as expected the reduction was much more severe at 1x[CO_2_] (Fig 1a). The soil moisture content was reduced at both [CO_2_], but the soil moisture content of -H_2_O stressed plants at 2x[CO_2_] was approximately 74% greater than corresponding plants as 1x[CO_2_] (Fig 1b). Under water limiting conditions (-H_2_O) the Pn in plants at 2x[CO_2_] was significantly higher than plants at 1x[CO_2_] (Fig 1c). Therefore, the imposed drought treatment was sufficient to stress both sets of plants, but also represent the physiological advantage provided by the interaction between elevated [CO_2_] and drought. Collectively, these results are consistent with results of others [5,38,40,41] and allow examination of additional biotic interactions in this system.

### Elevated [CO_2_] and drought enhanced maize susceptibility to *F*. *verticillioides* proliferation and fumonisin contamination

**Fig 2:**
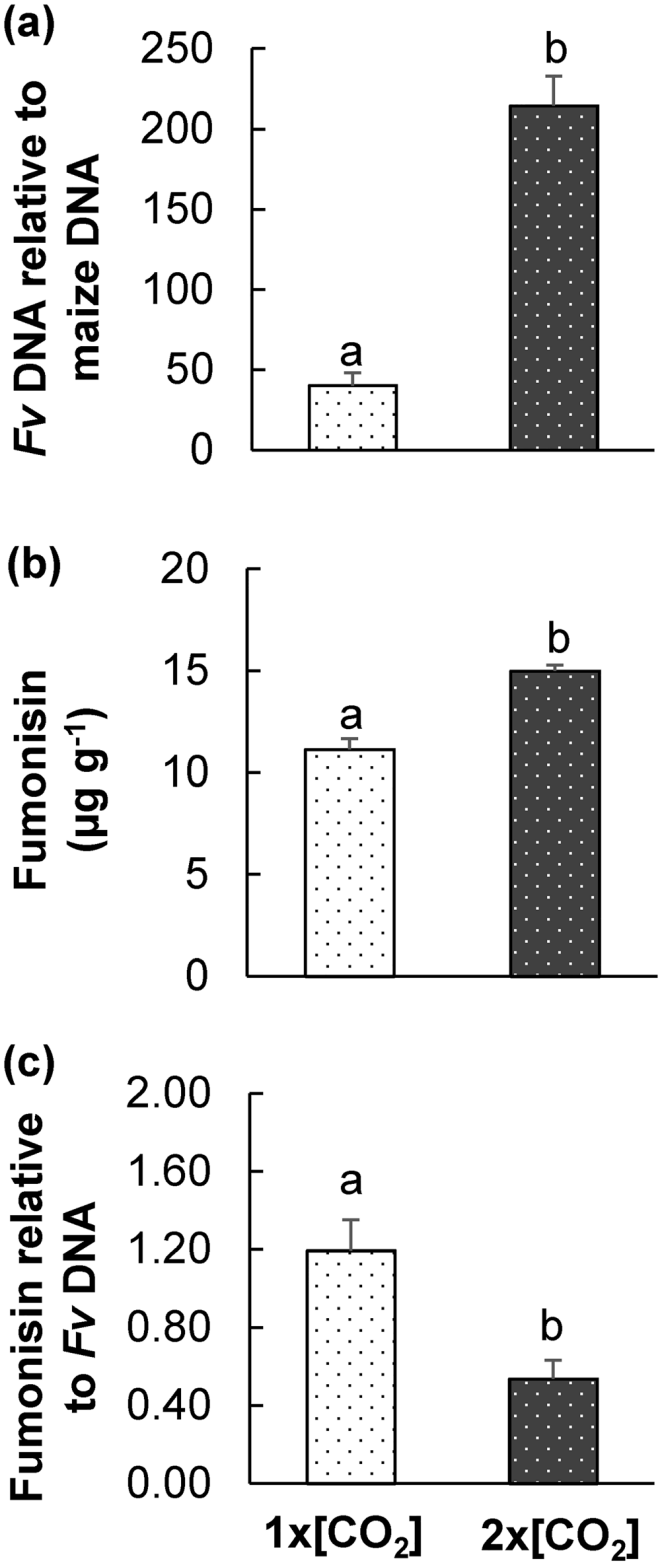
2x[CO_2_] and drought (-H_2_O) increased maize susceptibility to *F*. *verticillioides* (*Fv*) proliferation and fumonisin contamination. (a) The average *Fv* biomass in maize stalks seven days post-inoculation grown at 1x[CO_2_] or 2x[CO_2_] with drought (-H_2_O) was estimated as the amount of pg fungal DNA relative to ng maize DNA via qRT-PCR. (b) The mean concentration of fumonisin contaminating stalks grown under different abiotic stress conditions was determined, and (c) the relative amount of fumonisin per pg of *Fv* DNA was estimated. Values represent averages ± SEM. Letters above bars indicate significant differences (*t*-test, n = 4, *P*<0.05).

The combined effects of 2x[CO_2_] and -H_2_O on stalk rot were evaluated by quantifying the amount of pathogen biomass and fumonisin. As determined by quantitative real time-PCR (qRT-PCR) analysis, 7 d post-inoculation the *Fv* biomass in 2x[CO_2_]-H_2_O maize was 4-fold more than plants at 1x[CO_2_]-H_2_O (Fig 2a). The concentration of fumonisins was also significantly greater but only by 0.4-fold (*P*<0.01; Fig 2b). Therefore, the amount of fumonisins relative to fungal biomass was reduced in plants at 2x[CO_2_]-H_2_O (Fig 2c).

An ANOVA was also performed to evaluate mean differences with data acquired from 1x[CO_2_] and 2x[CO_2_] +H_2_O (data published in [11]). [CO_2_], H_2_O and the interaction between the two factors significantly contributed to differences in *Fv* biomass (*P*<0.01). 2x[CO_2_]-H_2_O+*Fv* plants contained 69% more *Fv* biomass than at 2x[CO_2_]+H_2_O+*Fv*, but 1x[CO_2_]-H_2_O+*Fv* maize had 38% less *Fv* biomass than plants at 1x[CO_2_]+H_2_O+*Fv*. Similarly, fumonisins levels were higher at 2x[CO_2_]-H_2_O+*Fv* but lower at 1x[CO_2_]-H_2_O+*Fv*. [CO_2_] was the only significant contributing factor to differences in the amount of fumonisins relative to fungal biomass.

### The interaction between elevated [CO_2_] and drought was not a contributing factor to changes in primary metabolite concentrations during *F*. *verticillioides* infection

**Fig 3:**
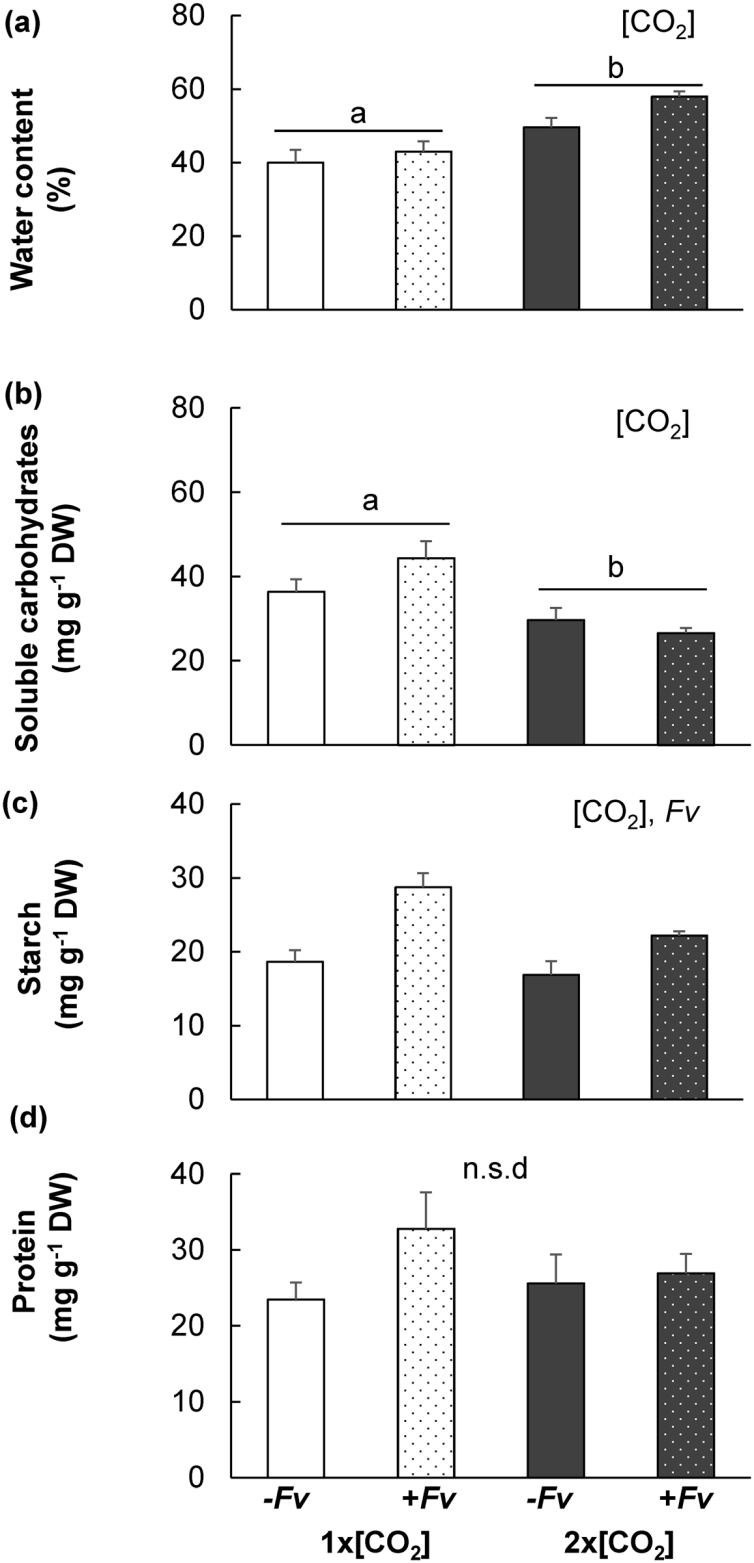
Influence of 2x[CO_2_] and drought (–H_2_O) on maize primary metabolite concentrations following *F*. *verticillioides (Fv)* inoculation. The average ± SEM (a) water content, (b) total soluble carbohydrates, (c) starch and (d) total protein in maize stems two days post mock-inoculation (-*Fv*) or *F*. *verticillioides* inoculation (+*Fv*) under different [CO_2_] and drought (–H_2_O) was evaluated and compared. Significant contributing factors to differences are indicated in the top right hand corner of each graph (2x2 ([CO_2_]x*Fv*), ANOVA, n = 4, *P*<0.05).

The water content in the stem tissues of plants under different abiotic stress treatments was determined so that all the metabolite concentrations could be reported on a dry weight basis. [CO_2_] was the main effect contributing differences in stem water content with -H_2_O, which is consistent with enhanced water-use efficiency at elevated [CO_2_] (Fig 3a).

Since previous results demonstrated a significant interaction between [CO_2_] and *F*. *verticillioides* pathogen infection (+*Fv*) on maize sugar levels [11], we evaluated the concentration of primary metabolites under conditions of -H_2_O. The induction in soluble carbohydrate concentrations with +*Fv* which was observed in 1x[CO_2_]+H_2_O [11] was not detected in -H_2_O plants at either 1x[CO_2_] or 2x[CO_2_] (Fig 3b). However, the concentration of starch was significantly increased by +*Fv* (Fig 3c). No significant difference was detected between protein concentrations of -H_2_O plants (Fig 2d).

**Fig 4:**
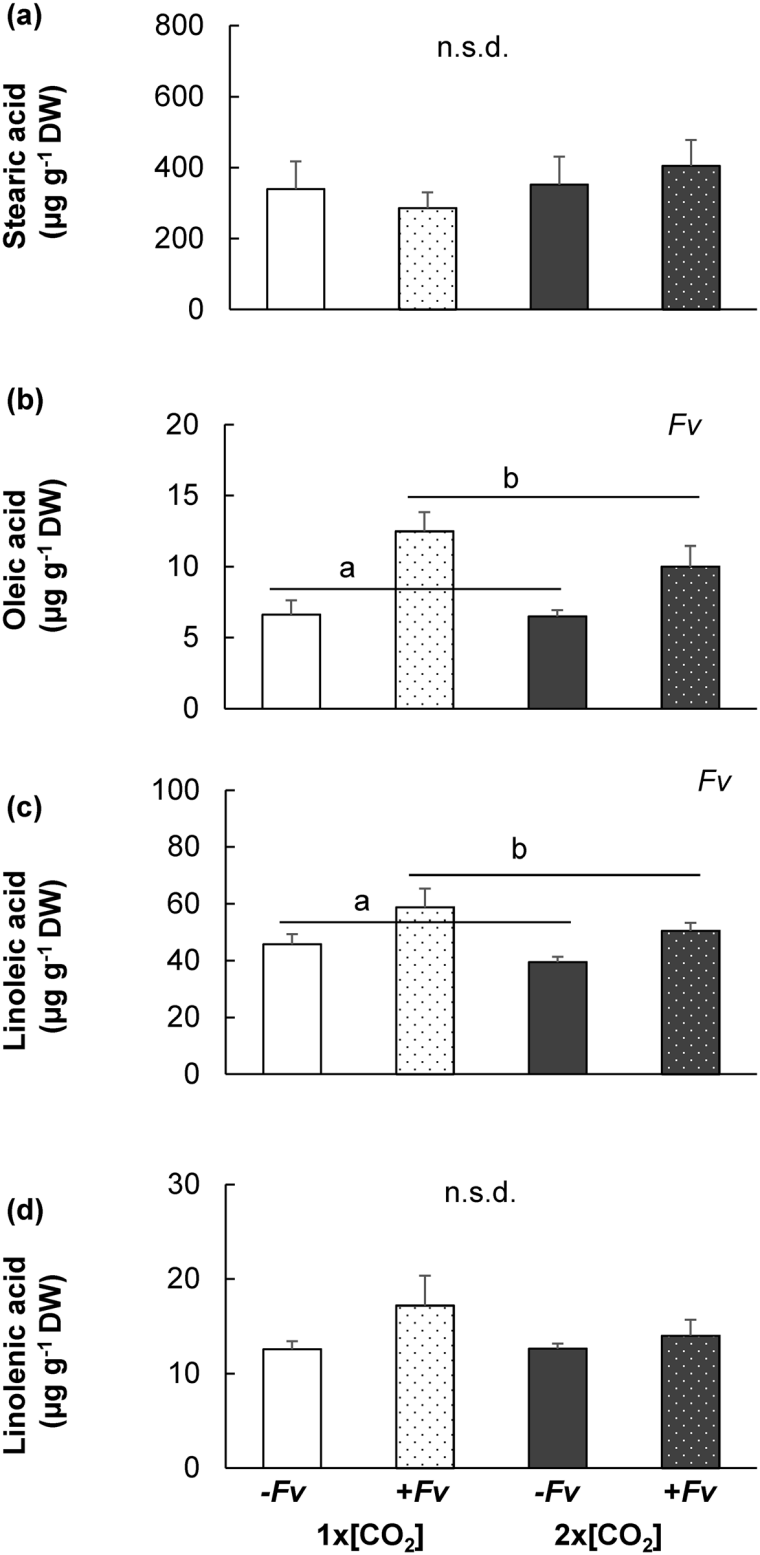
Influence of 2x[CO_2_] and drought (–H_2_O) on maize free fatty acid concentrations following *F*. *verticillioides* (*Fv)* inoculation. The average ± SEM concentration of (a) stearic acid, (b) oleic acid, (c) linoleic acid and (d) linolenic acid in maize stems two days post mock inoculation (-*Fv*) or *F*. *verticillioides* (+*Fv)* inoculation at 1x[CO_2_] or 2x[CO_2_] under conditions of drought (–H_2_O) was determined. Individual factors contributing to differences are indicated at the top right corner of each graph (2x2 ([CO_2_]x*Fv*) ANOVA, n = 4, *P*<0.05).

The concentrations of free fatty acids which are key precursors in oxylipin biosynthesis [13,18,42], were also analyzed to assess the combined influence of both elevated [CO_2_] and -H_2_O on maize response to +*Fv*. Under conditions of -H_2_O, only the concentration of oleic acid and linoleic acid were significantly induced by +*Fv* and this induction was not influence by [CO_2_] (Fig 4). However, in comparison to plants at 1x[CO_2_]+H_2_O+*Fv* [11] the induction of stearic acid, oleic acid, linoleic acid and linolenic acid in -H_2_O stressed plants at both [CO_2_] had significantly reduced levels of free fatty acids. According to the ANOVA model comparing irrigation treatments ([11] and Fig 4), H_2_O, [CO_2_], *Fv* and [CO_2_] x *Fv* were significant contributing factors for differences in fatty acid concentrations, with the exception of stearic acid which followed the same trend but no significant difference was detected. The concentration of linolenic acid in 1x[CO_2_]-H_2_O+*Fv* stems was approximately 40% less than the concentration in 1x[CO_2_]+H_2_O+*Fv* stems.

### Drought did not negate the compromising effects of elevated [CO_2_] on the accumulation of phytohormones following *F*. *verticillioides* inoculation

**Fig 5:**
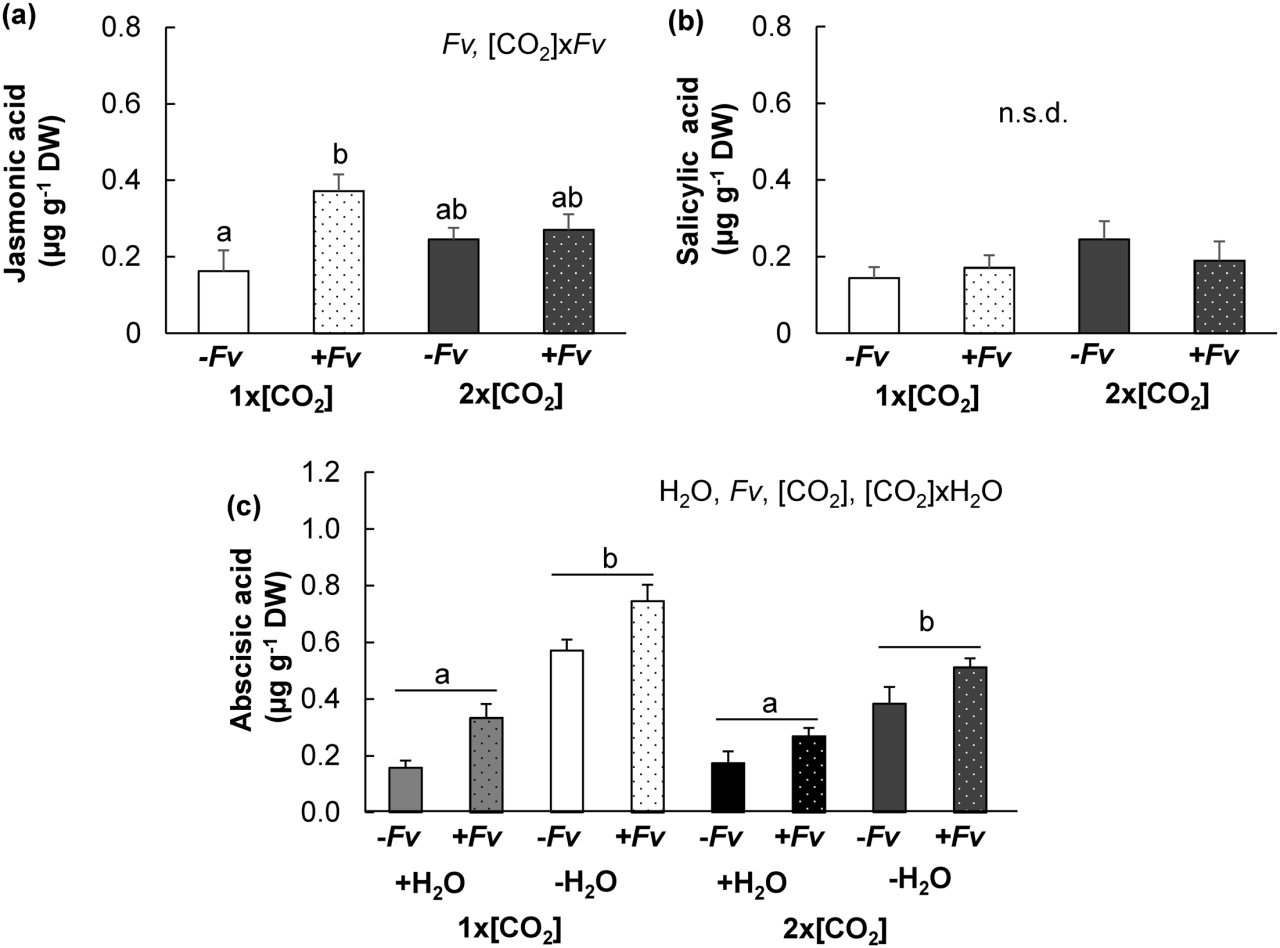
Effects of 2x[CO_2_] and drought (–H_2_O) on maize phytohormone responses to *F*. *verticillioides* (*Fv)* inoculation. Mean concentration ± SEM of (a) jasmonic acid (JA), (b) salicylic acid (SA), and (c) abscisic acid in maize stem tissues grown at 1x[CO_2_] or 2x[CO_2_] with irrigation (+H_2_O) or without irrigation (-H_2_O) conditions two days after mock inoculation (-*Fv*) or *F*. *verticillioides* inoculation (+*Fv*) were determined. Individual and interacting factors significantly contributing to differences are indicated at the top right corner of each graph (2x2x2 ([CO_2_]xH_2_Ox*Fv*) ANOVA, n = 4, *P*<0.05).

To evaluate the combined effects of 2x[CO_2_] and -H_2_O on the maize defense response, we analyzed the concentration of major phytohormones in stem tissues of -*Fv* and +*Fv* plants. Consistent with +H_2_O maize [11], the concentration of JA was induced 2 d post +*Fv* in maize at 1x[CO_2_]-H_2_O but not at 2x[CO_2_]-H_2_O (Fig 5a). However, there was no significant difference between the JA concentration of 1x[CO_2_]-H_2_O+*Fv* and 2x[CO_2_]-H_2_O+*Fv* plants. SA concentration displayed no significant difference among treatments with–H_2_O (Fig 5b), but in comparison to +H_2_O plants [11], -H_2_O plants had significantly lower levels of both JA and SA 2 d post inoculation. Furthermore, SA levels were not reduced with +*Fv* under conditions of–H_2_O (Fig 5) as observed with +H_2_O [11]. In contrast to the other phytohormones, ABA levels increased with drought and was highest in plants at 1x[CO_2_]-H_2_O (Fig 5c). Both [CO_2_] and [CO_2_] x H_2_O were significant factors contributing to differences in ABA concentrations (*P*<0.01) presumably due to the variable level of drought stress in plants at 1x[CO_2_] and 2x[CO_2_], which is consistent with the amelioration of drought stress at 2x[CO_2_]. Additionally, *Fv* inoculation stimulated the production of ABA at both [CO_2_] suggesting that 2x[CO_2_] does not dampen the induction of ABA.

**Fig 6:**
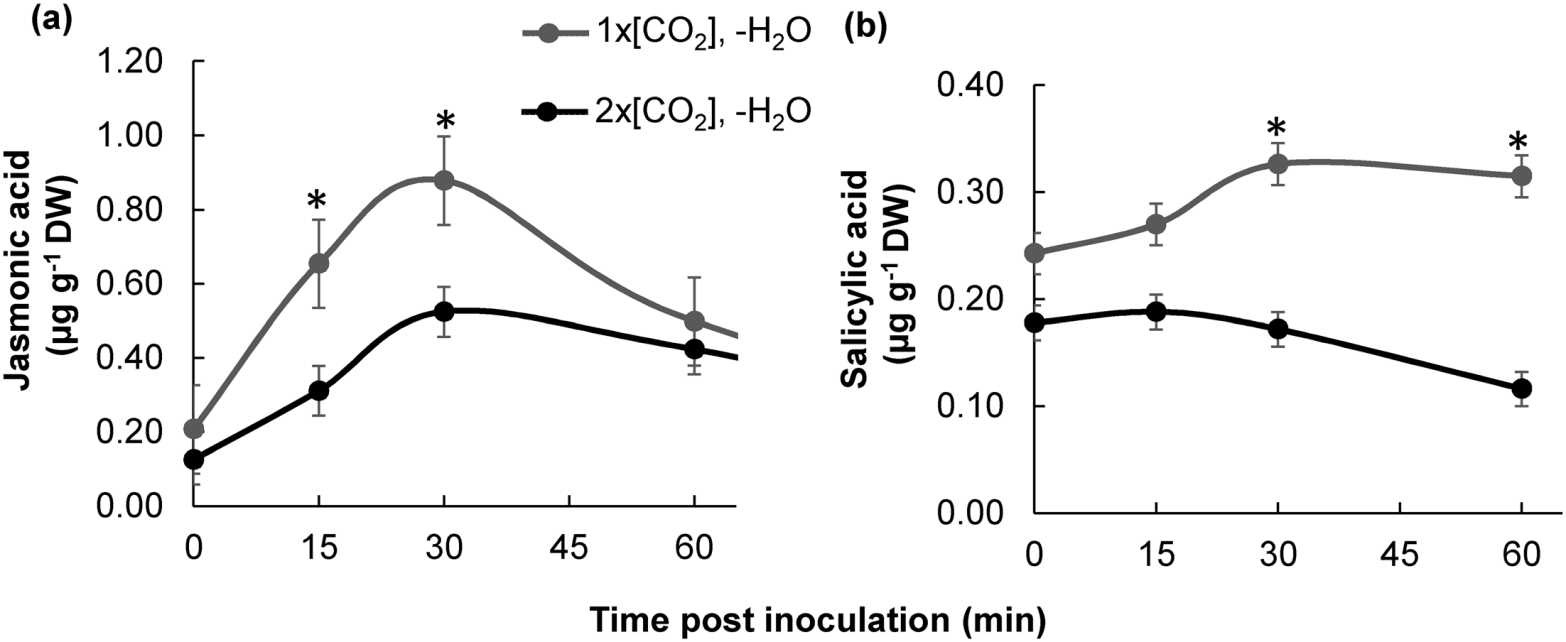
The initial burst of phytohormones following *F*. *verticillioides* (*Fv)* inoculation was reduced in plants under 2x[CO_2_] and drought (-H_2_O) conditions. The initial responses of (a) JA and (b) SA was tracked by quantifying phytohormone levels throughout a one hour time course post *Fv* inoculation of drought treated (-H_2_O) maize stalks at 1x[CO_2_] or 2x[CO_2_]. Error bars represent SEM and asterisks indicate significant differences between 1x[CO_2_] and 2x[CO_2_] at a particular time point (*t*-test, n = 4, *P*<0.01).

In order to further assess the combined effects of elevated [CO_2_] and drought on the initial burst of JA and SA, phytohormone concentrations were analyzed at several time points over a 1 h time course immediately following +*Fv*. In combination with -H_2_O, 2x[CO_2_] still dampened the early induction of JA (Fig 6a). The greatest difference of JA between 1x[CO_2_]-H_2_O+*Fv* and 2x[CO_2_]-H_2_O+*Fv* was observed at the 15 min time point when the concentration of JA in 1x[CO_2_]-H_2_O+*Fv* was double that of plants at 2x[CO_2_]-H_2_O+*Fv* (*P*<0.01). The induction of JA was more rapid and JA concentrations reached higher levels in -H_2_O as compared to +H_2_O plants ([11] and Fig 6a). The early accumulation of SA was also significantly dampened (Fig 6a). The concentration of SA in plants at 2x[CO_2_]-H_2_O was significantly less than 1x[CO_2_]-H_2_O at both the 30 min and 60 min time point (Fig 6b, *P*<0.05). At the time points evaluated, -H_2_O did not appear to significantly influence SA levels when compared to +H_2_O at the same [CO_2_] ([11] and Fig 6b).

### Elevated [CO_2_] and drought further dampened maize defense metabolism

The even greater susceptibility of maize to *Fv* proliferation at 2x[CO_2_]-H_2_O is consistent with a weaker response of important phytochemicals involved in resistance. Therefore, to evaluate the downstream impact of abiotic stress induced changes in phytohormone signaling, the concentrations of major maize defense metabolites were analyzed and compared.

**Fig 7:**
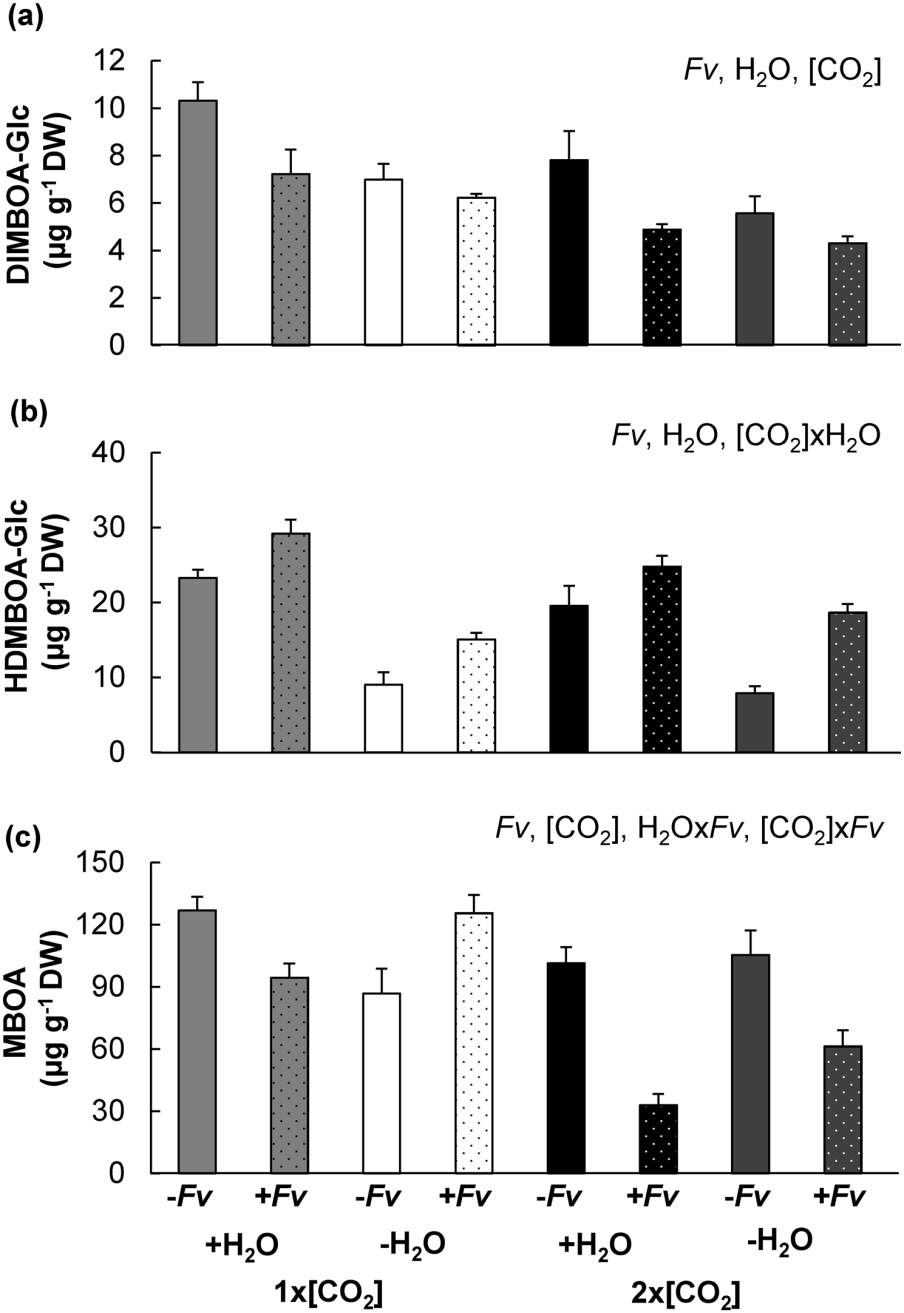
Effects of 2x[CO_2_] and drought (–H_2_O) on maize benzoxazinoid defense metabolites. The mean ± SEM concentration of (a) DIMBOA-Glc, (a) HDMBOA-Glc and the downstream degradation product of their aglycones, (c) MBOA was determined for both mock (-*Fv*) or *F*. *verticillioides* (+*Fv*) inoculated stem tissues. Individual and interacting factors significantly contributing to differences are indicated at the top right corner of each graph (2x2x2 ([CO_2_]xH_2_Ox*Fv*) ANOVA, n = 4, *P*<0.05).

Although the biologically inactive 2-(2,4-dihydroxy-7-methoxy-1,4-benzoxazin-3-one)-beta-D-glucopyranose (DIMBOA-Glc) and 2-(2-hydroxy-4,7-dimethoxy-1,4-benzoxazin-3-one)-beta-D-glucopyranose (HDMBOA-Glc) are synthesized during development and are primarily thought of as phytoanticipins, it has been suggested that JA is involved in the signal transduction leading to the conversion of DIMBOA-Glc into HDMBOA-Glc in response to pathogen attack [34,43,44]. Therefore, compromised JA signaling could also influence this conversion. The interaction between [CO_2_] and H_2_O did not influence DIMBOA-Glc concentrations. However, independent of other treatments, [CO_2_], H_2_O and *Fv* did significantly reduce DIMBOA-Glc concentrations (Fig 7a). According to the ANOVA model, the concentration of HDMBOA-Glc was significantly influenced by the interaction between [CO_2_]xH_2_O (Fig 7b). But the slight reduction of HDMBOA-Glc in stems at 2x[CO_2_]+H_2_O was only marginally significant (*P* = 0.06) in comparison to concentrations 1x[CO_2_]+H_2_O using the Tukey-Kramer test. No significant difference was detected in HDMBOA-Glc at 1x[CO_2_]-H_2_O and 2x[CO_2_]-H_2_O.

The unstable biologically active aglycones were not directly quantified, but the concentration of their degradation product MBOA was determined (Fig 7c). With the exception of plants at 1x[CO_2_]-H_2_O, the concentration of MBOA was reduced with +*Fv*. The concentration of MBOA appeared to follow the inverse pattern of *Fv* biomass. The highest concentration of MBOA coincided with the least amount of pathogen in 1x[CO_2_]-H_2_O plants and the lower concentrations of MBOA coincided with the higher amounts of pathogen in plants at 2x[CO_2_]. However, 2x[CO_2_]-H_2_O+*Fv* plants which had the greatest amount of *Fv* biomass contained more MBOA then 2x[CO_2_]+H_2_O+*Fv*.

**Fig 8:**
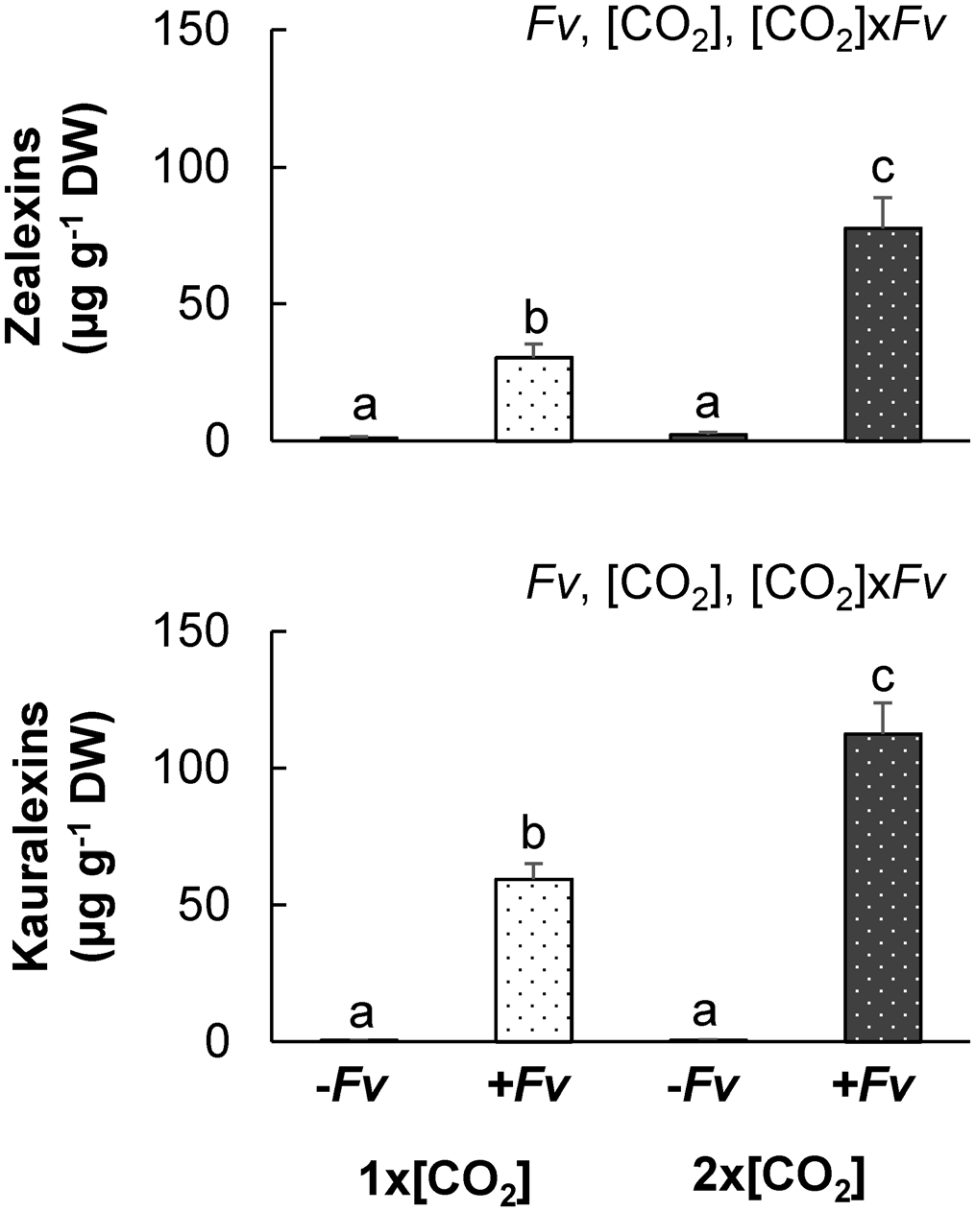
Effects of 2x[CO_2_] and drought (–H_2_O) on the accumulation of maize terpenoid phytoalexins following *F*. *verticillioides* (*Fv)* inoculation. Average concentration ± SEM of (a) total zealexins and (b) kauralexins in maize stalks grown at 1x[CO_2_] or 2x[CO_2_] with drought (-H_2_O) treatment two days after mock inoculation (-*Fv*) or *F*. *verticillioides* inoculation (+*Fv*) was determined. Statistically significant factors and interactions are included at the top right corner of each graph 2x2 ([CO_2_]x*Fv*) ANOVA. Since the interaction between the contributing factors was significant, a Tukey- Kramer HSD test was performed to determine differences between each mean. Letters above standard error of mean (SEM) bars indicate significant differences (n = 4, *P*<0.01).

Although the accumulation of terpenoid phytoalexins was significantly influenced by the interaction between [CO_2_] and *Fv* at–H_2_O, the concentration of zealexins and kauralexins was greater in 2x[CO_2_]-H_2_O+*Fv* plants than in 1x[CO_2_]-H_2_O+*Fv* (Fig 8) which is opposite of what was observed in corresponding +H_2_O plants [11]. Treatment with +*Fv* strongly induced the production of both zealexin and kauralexin families; however, the accumulation was significantly less in comparison to 1x[CO_2_]+H_2_O+*Fv* [11]. Terpenoid phytoalexin accumulation was weakest in stems at 1x[CO_2_]-H_2_O where the concentration of zealexins and kauralexins was approximately 34% and 22% the concentration in 1x[CO_2_]+H_2_O+*Fv* stems, respectively. Additionally, the induction of terpenoid phytoalexins was dampened in plants at 2x[CO_2_] regardless of H_2_O treatment. The concentration of total zealexins at 2x[CO_2_] was not further reduced by -H_2_O, but the amount of total kauralexins in +*Fv* stem tissues at 2x[CO_2_]-H_2_O was 25% less than the concentration in 2x[CO_2_]+H_2_O+*Fv* stems ([11] and Fig 8b).

### Elevated [CO_2_] does not influence drought induced accumulation of maize root terpenoid phytoalexins

**Fig 9:**
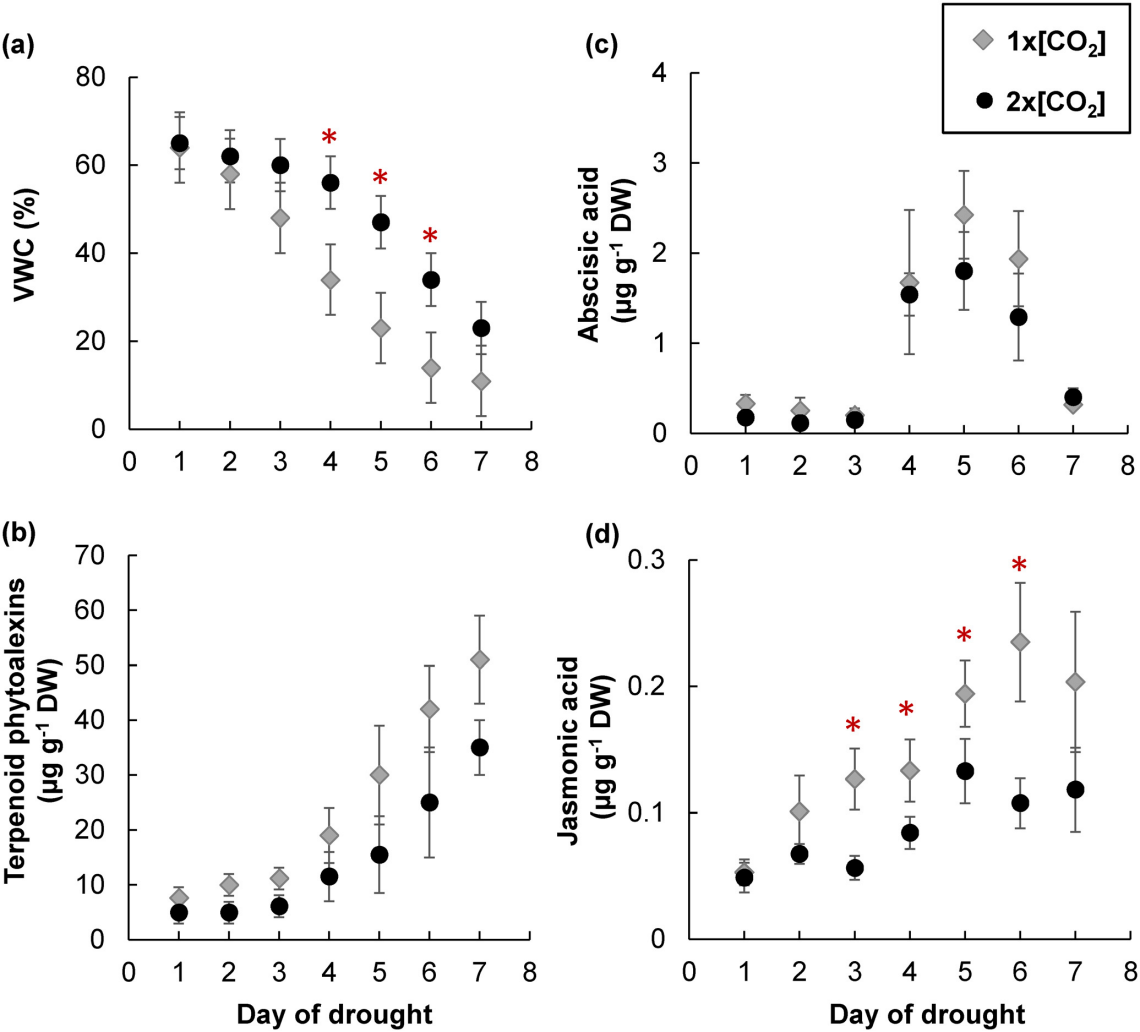
Elevated [CO_2_] does not inhibit the drought induced accumulation of maize root terpenoid phytoalexins. Water was withheld from V8 stage plants at 1x[CO_2_] and 2x[CO_2_] for seven consecutive days throughout which the (a) percent volumetric water content (VWC) was monitored using an EC-5 soil moisture sensor. Each day a subset of roots was collected for extraction and quantification of (b) total terpenoid phytoalexins (sum of both the zealexin and kauralexin metabolites), (c) abscisic acid, (d) jasmonic acid. Values represent the mean (± SEM) of five samples at each corresponding day of drought. The variable mean at 1x[CO_2_] and 2x[CO_2_] for each time point was compared independent of others using a Student’s *t*-test. Red asterisk indicate points at which values at 1x[CO_2_] and 2x[CO_2_] are significantly different (*P*<0.05).

Since the accumulation of terpenoid phytoalexins in drought stressed maize roots could potentially influence the induction potential in aboveground organs [28], the concentration of root terpenoid phytoalexins in plants grown at 1x[CO_2_] and 2x[CO_2_] was evaluated over a time course of withholding water. Even though the soil volumetric water content (VWC) of plants at 2x[CO_2_] did not decline as quickly as plants at 1x[CO_2_], the induction of terpenoid phytoalexins was not significantly different (Fig 9a and 9b). The spike of ABA was also not significantly different between the two [CO_2_] treatments (Fig 9c). However, the gradual increase in JA was dampened at 2x[CO_2_] (Fig 9d). At day 6 of withholding water, plant roots at 1x[CO_2_] contained approximately two times the amount of JA compared on to roots at 2x[CO_2_] (*P*<0.05).

**Fig 10:**
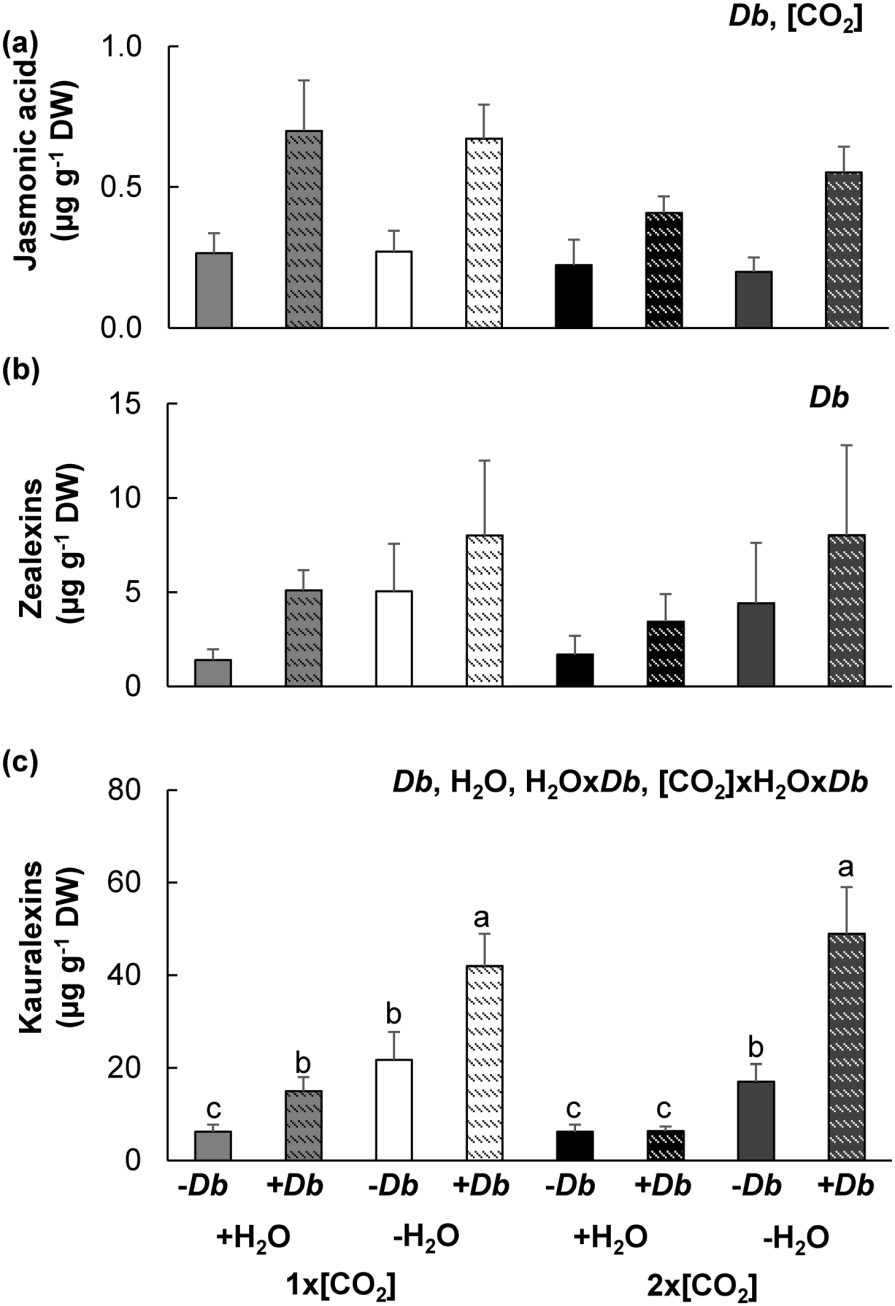
Drought negates the compromising effects of elevated [CO_2_] on the accumulation of root terpenoid phytoalexins to *Diabrotica balteata* feeding damage. The average concentration ± SEM of (a) jasmonic acid (b) zealexins and (c) kauralexins in roots of maize grown at 1x[CO_2_] or 2x[CO_2_], with irrigation (+H_2_O) or drought (-H_2_O) treatment, 2 d post control (-*Db*) or *Diabrotica balteata* larvae infestation (+*Db*) was determined. Statistically significant factors and interactions are included at the top right corner of each graph 2x2x2 ([CO_2_]xH_2_Ox*Fv*) ANOVA. If the interaction between the contributing factors was significant, a Tukey- Kramer HSD test was performed to determine differences between means (n = 4, *P*<0.05). If there was no interaction, a *t*-test was performed on the means of the main effect (n = 16, *P*<0.01). Letters above bars indicate significant differences.

To distinguish between the potential compromising effects 2x[CO_2_] on JA signaling in maize roots and the consequence of enhanced water-use efficiency at 2x[CO_2_], the concentration of the JA and terpenoid phytoalexins in *Diabrotica balteata* larvae infested (+*Db*) and control (-*Db*) roots grown under the different abiotic stress treatments was measured. The interaction between [CO_2_] and *Db* infestation was only marginally significant (*P* = 0.07; Fig 10a). At 2 d post infestation, JA levels increased with larval root feeding (*P*<0.01); however, the concentration of JA in root tissues at 2x[CO_2_] was still significantly less than roots at 1x[CO_2_] (*P*<0.01). While *Db* infestation was the only significant factor contributing to differences in root zealexin concentrations (*P*<0.05; Fig 10b), kauralexin concentrations were influenced by all three interacting factors [CO_2_]xH_2_Ox*Db* (*P*<0.01; Fig 10c). Kauralexin concentrations increased with *Db* feeding damage or -H_2_O stress, and simultaneous root feeding damage and drought made the accumulation of kauralexins even stronger. Irrigated maize exposed to simultaneous 2x[CO_2_]+*Db* did not display an increase in kauralexins. However, since the kauralexin concentration increased in maize roots with -H_2_O regardless of the compromising effects of 2x[CO_2_], there was no significant difference in root kauralexin levels between plants exposed to simultaneous 1x[CO_2_]-H_2_O+*Db* and 2x[CO_2_]-H_2_O+*Db*.

## Discussion

Our research has demonstrated that while the physiological effects of elevated [CO_2_] provide a photosynthetic advantage to maize under conditions of drought (-H_2_O), the plants are more susceptible to *Fusarium verticillioides* (*Fv*) proliferation and prone to higher levels of fumonisin contamination (Figs 1 and 2). Recent reviews have summarized the potential effects of future climate scenarios on the development of plant diseases and disease epidemics; however, there is a general consensus that relatively few studies have evaluated the interactive effects of multiple climate change factors on host-pathogen interactions, and further research is still required particularly with respect to mycotoxigenic pathogens [8,45–48]. This report represents an analysis of the interactive influence of elevated [CO_2_] and drought on maize phytochemical defense responses to an economically important mycotoxigenic pathogen. Our findings provide insight into climate change induced host metabolic alterations that lead to variation in crop susceptibility and mycotoxin contamination.

The majority of studies investigating the effects of climate change on plant defense are on C_3_ plants and plant-herbivore interactions [19,49,50]. Emerging results suggest that abiotic stress can modulate phytohormone signals leading to differential responses to biotic stress, variation in downstream defense chemistry, and changes in susceptibility [50]. This is consistent with our findings in C_4_ maize both the defense response to *Fv* stem inoculation and *Db* root feeding was altered by the abiotic stress treatments (Figs 2 and 10 and [11]). However, the dampened response of JA regulated defenses and increase in SA regulated defenses in C_3_ plants under elevated [CO_2_] are negated in combination with drought [19].

In maize, the accumulation of both JA and SA is compromised under elevated [CO_2_] and these effects persist even in combination with drought (Fig 2). Therefore, aboveground the effects of elevated [CO_2_] supersede any potential stimulation of JA defenses with -H_2_O (Fig 6a and [51]). The initial induction of JA following *Fv* inoculation was stronger in -H_2_O stressed plants (Fig 6a) in comparison to +H_2_O plants [11]; however, this stimulation did not last (Fig 5a). Other literature reports also suggest that, on its own, drought does not adequately stimulate JA defenses in aboveground maize tissues [52]. Therefore, the mechanism of phytohormone modulation by which elevated [CO_2_] and drought alters defenses in C_4_ maize is different from those previously described for C_3_ plants (i.e. soybean) and require additional research so that strategies can be developed to ensure the quality and safety of the future maize crop under climate change scenarios.

The increase in maize susceptibility to *Fv* proliferation suggests a compromised defense response. Consistent with this notion, the initial induction of JA and SA phytohormones was dampened at 2x[CO_2_]-H_2_O in comparison to 1x[CO_2_]-H_2_O (Fig 6). In comparison to 2x[CO_2_]+H_2_O plants though [11], JA levels of 2x[CO_2_]-H_2_O plants were only slightly reduced (Fig 6). An even weaker JA signal should have resulted in reduced accumulation of zealexins and kauralexins. Although terpenoid phytoalexin concentrations were greater in infected plants at 2x[CO_2_]-H_2_O in comparison to 1x[CO_2_]-H_2_O (Fig 8), it is important to consider that the accumulation of these phytoalexins has been shown to be dependent on the amount of pathogen inoculum [34]. Therefore, while the reduction in pathogen biomass could account for the corresponding reduction in terpenoid phytoalexins at 1x[CO_2_]-H_2_O in comparison to 1x[CO_2_]+H_2_O, the concentration of both zealexin and kauralexin metabolites could be further induced in 2x[CO_2_]-H_2_O plants, which had significantly more pathogen biomass. Nevertheless, despite the potential augmented induction of plants at 2x[CO_2_]-H_2_O+*Fv* in comparison to 2x[CO_2_]+H_2_O+*Fv*, only the concentration of kauralexins was significantly less while the concentration of zealexins remained unchanged. However since the pathogen biomass was greater, the accumulation of zealexins was likely also compromised at 2x[CO_2_]-H_2_O in comparison to 2x[CO_2_]+H_2_O.

The redirection of resources to maize roots may have contributed to the weaker response of terpenoid phytoalexins leading to intensified aboveground susceptibility to *Fv* proliferation between +H_2_O and -H_2_O plants at 2x[CO_2_] (Fig 8 and [11]). The 20 carbon diterpenoid kauralexins require more resources and accumulate to higher concentrations than the 15 carbon sesquiterpenoid zealexins in -H_2_O stressed maize roots (Fig 9 and [28]). This could potentially explain the greater effect on kauralexin levels in maize stems in comparison to zealexin levels (Fig 8 and [11]). Although JA was also reduced in maize roots (Fig 10a) and the accumulation of terpenoid phytoalexins in response to *Diabrotica balteata* larval feeding was impaired under conditions of 2x[CO_2_] (Fig 10b and 10c), ABA signaling was unaffected (Figs 5c and 9c) and the accumulation of drought induced terpenoid phytoalexins was not inhibited (Figs 9b, 10b and 10c). This is consistent with previous results demonstrating that application of ABA was sufficient to induce the accumulation of maize root zealexins and kauralexins [28]. Consequently, in contrast to aboveground tissues, -H_2_O induced ABA in maize roots can counterbalance the compromising effects of 2x[CO_2_] on the accumulation of zealexins and kauralexins. Therefore, at least with respect to root terpenoid phytoalexins, drought does negate the effects of elevated 2x[CO_2_].

Glycosylated benzoxazinoid concentrations were also influenced by the abiotic stress treatments; however, these changes do not appear to be correlated with increased susceptibility to *Fv* under conditions of 2x[CO_2_]+H_2_O or 2x[CO_2_]-H_2_O. The decrease in DIMBOA-Glc and corresponding increase in HDMBOA-Glc following *Fv* inoculation, which has previously been reported with *F*. *graminearum* maize stem infection [53], was not inhibited by 2x[CO_2_] (Fig 7a and 7b). The DIMBOA-Glc 4-O-methyltransferase which converts DIMBOA-Glc to HDMBOA-Glc is thought to be regulated by JA [43], but HDMBOA-Glc can also accumulate *de novo* in response to pathogen attack [54]. Moreover, even the compromised JA signal was adequate to stimulate HDMBOA-Glc accumulation. In contrast, drought considerably reduced the concentration of HDMBOA-Glc. Benzoxazinoids are suspected to be involved in drought stress tolerance because drought or belowground application of ABA can induce the concentration of DIMBOA in maize leaves [33,55]. Although the highly reactive aglycones were not quantified in this study, DIMBOA concentrations likely increased in maize stalks with drought and may have partly contributed to the reduction in pathogen biomass at 1x[CO_2_]-H_2_O. DIMBOA-Glc levels on the other hand may not be significantly altered as previously shown for ABA treated plants [56] which is consistent with our results. *Fv* is highly resistant to MBOA and has the ability to detoxify the compound by actively metabolizing it into *N*-(2-hydroxy-4-methoxyphenyl) malonamic acid (HMPMA) which is nontoxic [57,58]. Consistent with *Fv* detoxification of MBOA, the concentration of MBOA tended to inversely track pathogen biomass. However, this did not hold true at 2x[CO_2_] between +H_2_O and -H_2_O plants which would suggest additional interactive effects of 2x[CO_2_] and -H_2_O on benzoxazinoids metabolism. Further research investigating the effects of multiple climate change factors on maize benzoxazinoids and their derivatives in interaction with a pathogen more sensitive to these defense metabolites (i.e. *Fusarium graminearum*) will be necessary to fully understand the potential implications of the abiotic stress induced changes in maize benzoxazinoid dominated defenses.

Although the fumonisin contamination was significantly higher in infected stems under simultaneous conditions of 2x[CO_2_] and -H_2_O, the amount of fumonisin per unit *Fv* biomass was reduced compared to +*Fv* maize at 1x[CO_2_]. These data are consistent with previous results indicating that elevated [CO_2_] compromises the transcriptional response of many of the 9- and 13-lipoxygenase (LOX) and their signaling products [11], which have the potential to stimulate mycotoxin production [59]. Even though the transcript levels of LOX genes were not measured in this study, the metabolite analysis supports this notion. The addition of drought did not ameliorate the effects of elevated [CO_2_] on the influx of fatty acid substrate needed for oxylipin biosynthesis, nor did it negate the dampened accumulation of the 13-LOX oxylipin JA following *Fv* infection (Figs 4–6). Nevertheless, even though host-derived mycotoxin stimulants are potentially still reduced, the even larger amount of *Fv* biomass on maize at 2x[CO_2_]-H_2_O was ample to lead to greater fumonisin levels and could therefore be an agriculturally relevant food safety concern.

Considering that the drought treatment imposed in these experiments was specifically designed to account for the physiological changes in water utilization at 2x[CO_2_], it is possible that, in comparison to a more moderate drought stress treatment at 1x[CO_2_], the amount of fumonisin contamination would be higher instead of lower. Numerous reports indicate that drought enhances maize susceptibility to *Fv* and fumonisin [9,60,61]; however, in these experiments at 1x[CO_2_]-H_2_O plants displayed both less *Fv* biomass and fumonisin compared to irrigated plants. Since maize utilizes more water at 1x[CO_2_] and the degree of drought stress was higher (Fig 1b), the percentage of water in the stem tissues was significantly less (Fig 3a). Therefore, it is likely that this lower water activity was not conducive to fungal growth [62] and reduced *Fv* biomass and fumonisin production. While normalizing for soil moisture content would provide additional insight into the effects of [CO_2_] at variable levels of drought, this is beyond the scope of the current manuscript. The difference in soil water content was a consequence of the plant’s physiological response to elevated [CO_2_] which was a factor being studied and was thus intentionally not controlled in these experiments. Elevated [CO_2_] has the potential to ameliorate the severity of drought; therefore, it is essential to understand how these abiotic factors will interact and influence *Fv* infection in comparison to conditions which will not receive this same benefit of water conservation. Timing of infection will likely also play an important role in fumonisin contamination levels as during episodes of drought maize at 2x[CO_2_] may also provide a more favorable environment for pathogen growth, allowing for a prolonged period of mycotoxin accumulation. Furthermore, while the chamber based studies provide valuable data in understanding the defense response under controlled conditions of biotic stress, there are multiple limitations including light intensity, breadth of spectral wavelength, and hindered root establishment. These factors likely contribute to the abiotic stress imposed and influence resource availability and distribution. Additional laboratory studies coupled with field based free air gas concentration enrichment (FACE) experiments are needed to determine the tradeoffs between the photosynthetic advantage of water conservation and increased susceptibility on maize grain productivity during simultaneous conditions of elevated [CO_2_] and drought. Nonetheless, given the heightened climate change concerns and the potential consequences of our uncertainties for future agricultural maize production, our findings have provided a foundation for additional research necessary for the development of climate resilient mycotoxin control strategies.
